# Supplementary material for: Asymmetric Synthesis of Quaternary Hydantoins via a Palladium-Catalyzed Aza-Heck Cyclization
Source: J Am Chem Soc. 2025 Nov 14;147(49):44692–8. doi: 10.1021/jacs.5c16022 (PMC12703750; doi:10.1021/jacs.5c16022)
Supplement: Supplementary file 2 [file ja5c16022_si_002.zip › All NMR FID Files/14/14_ProtonNMR/1/pdata/1/email_EM05-117-1-1H-MeCN_1_1.pdf]

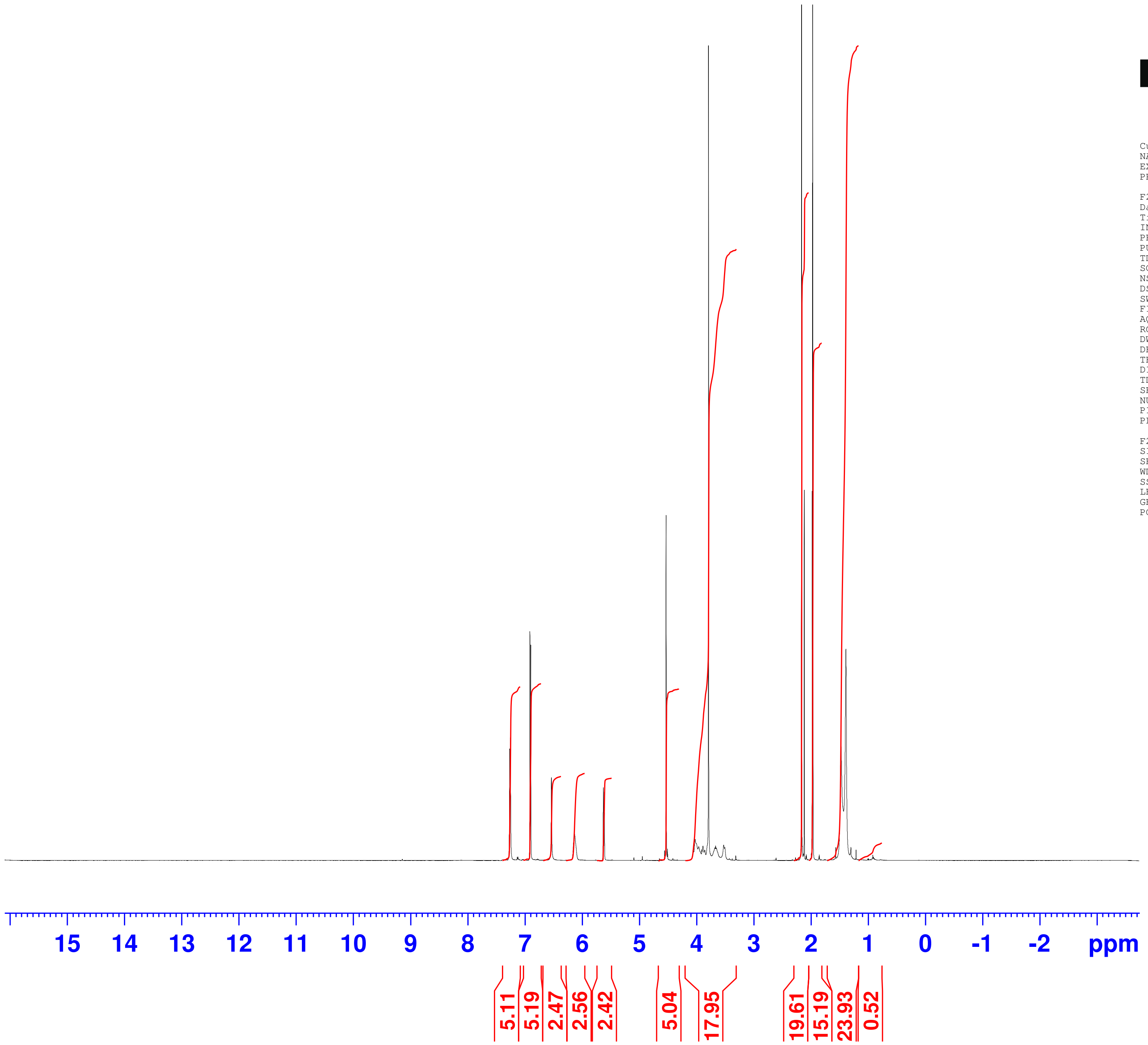

BRUKER

Current Data Parameters

NAME	EM05-117-1-1H-MeCN
EXPNO	1
PROCNO	1

F2 - Acquisition Parameters

Date_	20240423
Time	16.24 h
INSTRUM	Avance Neo 600 at University of Delaware
PROBHD	Z185170_0001 (
PULPROG	zg
TD	65536
SOLVENT	CD3CN
NS	16
DS	2
SWH	11904.762 Hz
FIDRES	0.363304 Hz
AQ	2.7525120 sec
RG	12.7
DW	42.000 usec
DE	10.66 usec
TE	298.0 K
D1	10.00000000 sec
TD0	1
SFO1	600.1337058 MHz
NUC1	1H
P1	8.00 usec
PLW1	6.90000010 W

F2 - Processing parameters

SI	65536
SF	600.1300000 MHz
WDW	EM
SSB	0
LB	0.30 Hz
GB	0
PC	1.00
